# Comment on Sifakaki et al. Orthorexia Nervosa Practices in Rheumatoid Arthritis: The DORA Study. *Nutrients* 2023, *15*, 713

**DOI:** 10.3390/nu15081984

**Published:** 2023-04-20

**Authors:** Adrian Meule

**Affiliations:** 1 Department of Psychiatry and Psychotherapy, University Hospital, LMU Munich, Nußbaumstr. 7, 80336 Munich, Germany; ameule@med.lmu.de; 2 Schoen Clinic Roseneck, Am Roseneck 6, 83209 Prien am Chiemsee, Germany

In a recently published article, Sifakaki and colleagues [1] conclude that patients with rheumatoid arthritis who receive medical nutrition therapy show orthorexic tendencies (i.e., a pathological preoccupation with healthy, “pure” eating that is associated with restrictive dietary patterns and nutritional deficiencies). This commentary argues that this conclusion is unwarranted because the authors used the ORTO-15 questionnaire [2] to assess orthorexic tendencies. However, numerous researchers have strongly advised against using the ORTO-15 questionnaire because it is psychometrically unsound, as it possesses low reliability and some items lack face validity [3,4,5,6,7].

The original scoring procedure of the ORTO-15 questionnaire [2] is quite peculiar; while all items are answered on a four-point scale with 1 = *always*, 2 = *often*, 3 = *sometimes*, and 4 = *never*, responses to four items then need to be inversely coded (i.e., 1 = 4, 2 = 3, 3 = 2, and 4 = 1), and two items are recoded as 2 = *always*, 4 = *often*, 3 = *sometimes*, and 1 = *never*. Sifakaki and colleagues [1] state that “the total score is calculated by summing the scores of the 15 individual items” (p. 3). However, this is obviously not how the questionnaire is scored according to Donini and colleagues [2]. Yet, irrespective of whether the authors used the scoring procedure proposed by Donini and colleagues [2] or summed up the raw item responses, neither of these procedures result in meaningful scores [5]. Furthermore, the authors used a Greek version of the ORTO-15 questionnaire, citing an article by Gkiouras and colleagues [8], in which it is explicitly stated that a one-factor model had poor model fit, with some items having factor loadings close to zero, indicating that the sum score of all 15 items should not be calculated, let alone interpreted.

This commentary piece is not the first advising against the use of the ORTO-15 questionnaire. In fact, this has been advised for many years now [3,4,5,6,7] and other measures for assessing orthorexic tendencies have been developed that are psychometrically sound [9]. While some of these alternatives may have limited suitability in clinical practice as they have more items than the ORTO-15 questionnaire and include subscales (e.g., the Eating Habits Questionnaire, the Teruel Orthorexia Scale, or the Orthorexia Nervosa Inventory), others, such as the Düsseldorf Orthorexia Scale [10], are well suited to clinical practice because of their briefness (10 Items), ease of scoring (all items are simply summed to a total score without the need to inversely code the items), and suggested cut-off scores. Because of the general issues related to the ORTO-15 questionnaire and the version used in the study by Sifakaki and colleagues, in particular, scores of this scale should not be interpreted and, therefore, it should not be concluded that patients with rheumatoid arthritis who receive medical nutrition therapy show orthorexic tendencies.
